# Current Advances in the Development of Decellularized Plant Extracellular Matrix

**DOI:** 10.3389/fbioe.2021.712262

**Published:** 2021-07-21

**Authors:** Yiwei Zhu, Qi Zhang, Shengyu Wang, Jianfeng Zhang, Shunwu Fan, Xianfeng Lin

**Affiliations:** ^1^Department of Orthopaedic Surgery, Sir Run Run Shaw Hospital, Medical College of Zhejiang University, Hangzhou, China; ^2^Department of Orthopaedics, The First Affiliated Hospital of Wenzhou Medical University, Wenzhou, China

**Keywords:** decellularized, plant, extracellular matrix, biocompatibility, biomaterials

## Abstract

An imbalance exists between the supply of organs for transplantation and the number of patients in the donor transplant waiting lists. Current use of autologous, synthetic, and animal-derived grafts for tissue replacement is limited by the low availability, poor biocompatibility, and high cost. Decellularized plant scaffolds with remarkable physical similarities to human organs have recently emerged and have been found to present favorable characteristics that make them suitable as an alternative biomaterial, such as a superficial surface area, excellent water transport and retention, pre-existing vascular networks, interconnected porosity, and a wide range of mechanical properties. In addition to their unique and superior biocompatibility, plant-derived scaffolds present the advantages of low production cost, no ethical or supply constraints, simple operation and suitability for large-scale production and research. However, there are still some problems and deficiencies in this field, such as immature decellularization standards and methods, insufficient research on the biocompatibility of plant extracellular matrix. At present, research on decellularized plant extracellular matrix is still in its infancy, and its applicability to tissue engineering needs to be further improved. In this review, the current research progress on decellularized plant scaffolds is reviewed, the problems to be solved and future research directions are discussed.

## Introduction

More than 200,000 patients need an organ transplant, while only about 15% of them received a transplant. And this disparity between organs and patients who need a donor organ remains one of medicine’s challenges (Author Anonymous, 2021). With the development of research, many materials from natural or artificial sources have been applied in transplantation, among which decellularized materials are considered as a promising biomaterial. At present, decellularized animal scaffold is the most widely used, in which all the cells are removed by physical, chemical or biological methods, and the components of extracellular matrix are retained as much as possible. Then, acellular scaffolds can be reseeded with human cells to produce an autologous graft (Badylak et al., 2009). However, decellularized animal scaffolds bring some problems, such as low production, high cost and ethical issues (Huerta et al., 2016).

Recently, plant scaffolds have been widely studied as substitutes for animal acellular scaffolds. In 2014, apple hypanthium tissue was made into a natural cellulose fiber scaffold by decellularization because of its special internal structure composed of pores and air pockets, which supports growth of mammalian cells *in vitro* for 12 weeks (Modulevsky et al., 2014). In 2017, decellularized spinach leaves were obtained by retaining their vascular structures, which resemble mammalian vasculature (Gershlak et al., 2017). Since then, various plant-based tissues, including parsley, bamboo, *Calathea zebrina*, *and Anthurium warocqueanum*, have been decellularized as scaffold materials (Fontana et al., 2017). Because of the existence of a variety of tissue structures in the plant kingdom, plant-derived acellular scaffolds can simulate human organs according to the structural characteristics of plants (Gershlak et al., 2017). In addition to preserving the characteristics of animal structures, plant structures are characterized by their unique and biological compatibility, low production cost, freedom from ethical and supply constraints, simple operation and are suitable for large-scale production and research (Gershlak et al., 2017). The modifiability and complete vascular system of plant-based tissues make them suitable for the growth of a variety of stem cells, and they present good prospects for use in heart regeneration (Gershlak et al., 2017) and bone tissue regeneration (Lee et al., 2019). However, there are still many problems with decellularized plant scaffolds, such as immunogenicity, biodegradability, immature decellularization standards, and methods. In this review, the current research progress of decellularized plant scaffolds is reviewed, and the existing problems and the future research directions are discussed.

## The Extracellular Matrix in Plant

The plant cell is the fundamental unit of structure and function of plant life, which is composed of protoplast and cell wall (Caffall and Mohnen, 2009). Protoplast is the general term for all substances in the cell wall and is mainly made up of cytoplasm, nucleus, cell membrane and organelle. In addition, the plant cell wall, also regarded as the extracellular matrix of plants, form the shape of plant tissues and organs, and plays an important role in intercellular communication and plant-microbe interactions. The plant cell wall is mainly made up of polysaccharides, of which cellulose is the major component (Caffall and Mohnen, 2009). Cellulose is organized into para-crystalline structures, which embedded in a rich polysaccharide matrix, including hemicelluloses and pectins, as well as structural lignin and structural glycoproteins in certain plants (Zablackis et al., 1995).

Cellulose is a kind of long-chain polysaccharide composed of 7,000–15,000 glucose monomer units and is rotated 180° alternately, which is the major structural fiber in the plant. Cellulose molecules are arranged into microfibres with a diameter of approximately 3–4 nm (Hori et al., 2002; Peura et al., 2008; Fernandes et al., 2011). Cellulose has been widely used in regenerative medicine, such as cartilage (Müller et al., 2006; Wegst et al., 2015), bone (Liuyun et al., 2009; Zaborowska et al., 2010), and wound healing (Czaja et al., 2006, 2007). After immersion in Ca(OH)_2_ solution, the cellulose was covered with a calcium phosphate layer, which is considered to be a novel scaffold structure for cartilage tissue engineering (Müller et al., 2006). Liuyun et al. (2009) used a freeze-drying method to make nano hydroxyapatite, chitosan, and carboxymethyl cellulose polymer into a new biodegradable composite scaffold material, which has a good prospect in the field of bone regeneration.

Xylan is a kind of heterogeneous polysaccharide found in the cell wall of plants. Xylan has been proved to enhance the phagocytic capacity of macrophages and induce lymphocyte proliferation, which is related to the initial inflammation after fracture injury (Zhou et al., 2010; Fang et al., 2012). Bush et al. (2016) combined the xylan (hemicellulose) and chitosan to form composite hydrogel, which is suitable for repairing large bone defects as a bone graft substitute.

Lignin is a biopolymer with a three-dimensional network structure formed by the interconnection of three phenylpropane units through ether bonds and carbon–carbon bonds, which mainly located between cellulose fibers (Ponnusamy et al., 2019). Lignin shows good antioxidant activity and excellent mechanical properties. Zhang et al. (2019) combined lignin, chitosan, and polyvinyl alcohol to form composite hydrogel, which has better antioxidant activity and scavenging ability of free radicals, and can accelerate wound healing in mice.

Since there is a wide variety of plant structures in the plant kingdom, decellularized plant- derived scaffolds can be selected according to their own structure and properties to simulate the diversity of mammalian tissues.

## Overview of Native Plant Decellularization Protocols

**FIGURE 1:**
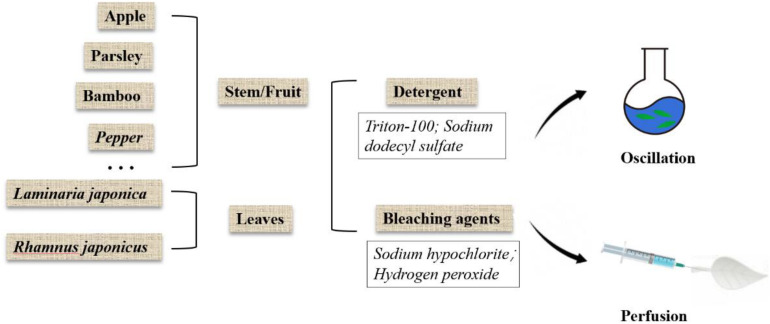
Overview of plant decellularization methods.

In the process of decellularization, the cellular components of the native plant tissue are removed and the decellularized scaffold is obtained. Ideally, all cellular components should be removed, while maximizing the preservation of plant extracellular matrix, as well as the plant’s structural and mechanical properties. In addition, toxic agents used in the decellularization regimen should be removed to ensure the biocompatibility of the scaffold. The existing decellularization protocols are shown in Table 1 and brief overview of the decellularization process is shown in Figure 1. Plant cuticle wax consists of a layer of white crystals covering on the surface of land plants, which inhibits the water loss from the surface of plants and protects plants from pathogen invasion. It is a hydrophobic organic mixture composed of superlong chain fatty acids and their derivatives, including alkanes, primary alcohols, and ketones, which can be extracted by using organic solvents. Therefore, in the process of decellularization of some plant tissues, hexane is used to remove the epidermal wax (Gershlak et al., 2017).

**TABLE 1:** Representative protocols used for plant decellularization.

Number	Decellularization method	Tissue type	Material size	References
➀	10% sodium dodecyl sulfate (SDS) for 5 days 1% non-ionic surfactant in 10% bleach solution until visibly cleared (visual comparison)	*F. hispida*	8 mm disk samples	Adamski et al. (2018)
➁	5% NaClO and 3% NaHCO_3_ until visibly cleared (visual comparison)	*F. hispida*	8 mm disk samples	Adamski et al. (2018)
➂	10% sodium dodecyl sulfate (SDS) for 5 days clearing solution (0.1% TritonX-100 and 10% sodium chlorite) for 2 days	Spinach and parsley	1 cm squares	Gershlak et al. (2017)
➃	10% sodium dodecyl sulfate (SDS) in water for 5 days 0.1% Triton-X-100 in a 10% solution of bleach for 2 days	*Calathea zebrina*, *Anthurium warocqueanum*, *Anthurium magnificum*, Solenostemon, Vanilla, Bamboo	8 mm disk	Fontana et al. (2017)
➄	0.5% sodium dodecyl sulfate (SDS)	Apple, Broccoli, Sweet pepper, Carrot, Persimmon, and Jujube	1 × 1 cm	Lee et al. (2019)
➅	1 mg/mL DNase I for 30 mins	Tobacco BY-2 cells, Rice cells Tobacco hairy roots	\	Phan et al. (2020)
➆	ScCO_2_ and 2% peracetic acid (PAA)	Spinach leaves, Parsley stems and Celery stalks	\	Harris et al. (2021)

*The decellularization protocol of different plants can be adjusted based on the existing protocols according to the size and shape of plants.*

### Detergent

The porous structure of most plants is conducive to decellularization because it allows rapid exchange of detergents, buffers, and media without the use of a perfusion system. Therefore, the decellularized cellulose scaffold can be made quickly by using only detergent. SDS is an anionic detergent that dissolves the cell membrane and nuclear membrane by breaking the bonds between the cell membrane and cytoplasmic proteins (Johnson, 2013). Because of the minimal physical agitation, SDS is likely to be suitable for most plant species, especially for cleaning entire leaves (Adamski et al., 2018).

### Bleaching Agents

Previous research has shown that leaves simmered in a solution of bleach (such as NaClO) and sodium bicarbonate help to separate vascular tissue from soft tissue, which carried out in the 17th and 18th centuries (Seba, 1730). These experiments soak plant tissues, such as leaves and fruits, in water until the soft tissues decompose naturally. This method is suitable for fragile tissue because it avoids extensive damage to the soft tissue structure. It has also been reported that this method has less effect on cell growth than the detergent-based method (Gershlak et al., 2017).

### Enzyme

Nuclease, which has been widely used in the process of animal tissue decellularization, provide high specificity in removing genetic material. Recently deoxyribonuclease (DNase) was firstly used in tobacco and rice (Phan et al., 2020). Phan et al. decellularized lyophilized tobacco and rice cells with 1 mg/mL DNA enzyme, and detergent method (0.25% sodium dodecyl sulfate or 0.1% Triton X-100) was used as control to investigate the effect of different surfactant treatments on DNA removal and protein retention. Result showed that plant cells and tissue can be completely decellularized with DNase, with substantial protein retention (Phan et al., 2020).

### Supercritical Carbon Dioxide

Supercritical carbon dioxide (ScCO_2_) refers to the special state where carbon dioxide behaves as neither a gas nor a liquid under critical conditions (temperature = 31.26°C, pressure = 27.9 atm). ScCO_2_ has been widely used in food industry, biology, pharmacy, and other fields because of its mild reaction conditions, non-toxicity, residue free reaction process, and easy operation (Guler et al., 2017). The mechanism of supercritical carbon dioxide for decellularization is still controversial. The hypothesis that high pressures burst the cells during treatment and cell removal was achieved by rapid depressurization has not been widely accepted and has been largely proved false. Harris et al. supposed that the pressure dissolves the carbon dioxide into the liquid phase, which allows it to penetrate the walls and membranes of plant cells. This transfer of ScCO_2_ reduces the intracellular pH through the formation of carbonic acid, which leads to disruption of cell metabolism and removal of essential enzymes. In addition, the combined application of supercritical carbon dioxide and co-solvents (like peracetic acid) can significantly improve the efficiency of decellularization (Harris et al., 2021).

## Mechanical Properties and Structures of Decellularized Plant Scaffolds

**FIGURE 2:**
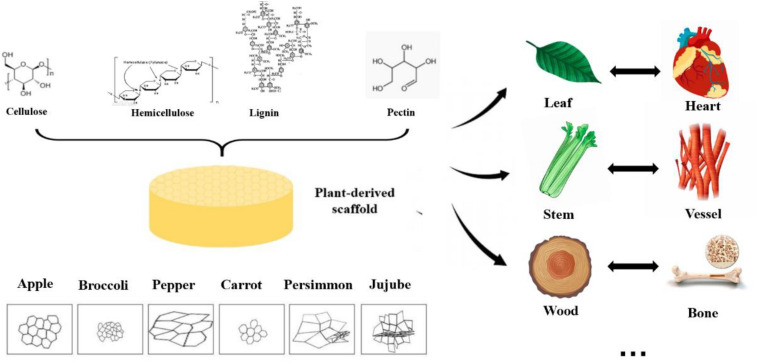
Structure, pore size and similarities between human organs and plant scaffolds: Plant scaffolds are basically composed of cellulose, hemicellulose, lignin and pectin, and the porosity size and pore shape of different plant tissues are quite different. A scaffold with potential for organ regeneration can be obtained by selecting the plant tissue that is similar to the human organ tissue in physical properties for decellularization treatment.

Due to the diversity of plant species in nature, their mechanical strength varies greatly, and some of them are highly similar to human tissues and organs in structure, shown in Figure 2. Some highly vascularized plant tissue, such as spinach leaves, may be more suitable for highly vascularized tissue (Gershlak et al., 2017), such as heart tissue, while the cylindrical hollow structure of plant stems may be more suitable for arterial transplantation. In addition, the high mechanical strength and geometric shape of wood make it a good application prospect in bone engineering.

### Mechanical Analysis of Decellularized Plant Scaffolds

The compositions of these different polysaccharides depend on the types of plant tissue, which endow them with specific physical properties. The modulus and compressive strength of plant-based materials span the whole range. The following Table 2 shows the mechanical properties of common plant decellularized scaffolds. The compression modules of decellularized apple tissue were similar to that of human adipose tissue, such as breast (2 kPa) and abdominal tissue (3 kPa). The compression modulus of carrot-derived scaffolds was 43.43 kPa, comparable to those scaffolds that have been widely proposed for non-load bearing bone scaffolds (Peng et al., 2019; Contessi Negrini et al., 2020). Hence, carrot-derived decellularized scaffolds may be applied to bone fillers under non-weight-bearing conditions. Decellularized celery was able to withstand 20% strain without failure, which is similar to maximum deformation value of natural tendon *in vivo* (Mathew et al., 2012; Contessi Negrini et al., 2020). In addition, the directional morphology of celery tissue has a strong resemblance with natural tendon tissue. Although decellularization process reduces the mechanical properties of plant tissue, they are still within the range of natural human tissues.

**TABLE 2:** Mechanical properties of existing plant scaffolds.

Plant type	Elastic modulus (kPa)	Stiffness (kPa)	Residual strain (%)	Maximum stress (kPa)
*Apple*_*decellularized*_ Contessi Negrini et al., 2020	4.17	4.33	6.42	1.17
*Apple*_*control*_ Contessi Negrini et al., 2020	4.36	9.47	6.48	2.07
*Carrot*_*decellularized*_ Contessi Negrini et al., 2020	43.43	/	/	44.31
*Carrot*_*control*_ Contessi Negrini et al., 2020	83.48	/	/	51.49
*Celery*_*decellularized*_ Contessi Negrini et al., 2020	594.78	/	/	175.93
*Celery*_*control*_ Contessi Negrini et al., 2020	552.49	/	/	174.60

### Pore Sizes of Plant Scaffolds

For pore size, 200–250 μm was the most suitable pore size for the growth and adhesion of human cells. The pore sizes of different plants vary greatly (from 20 to 800 mm), and are shown in Table 3. Before and after the process of decellularization, the changes of pore size of different types of plants are different, which may be related to the mechanical strength of plants (Lee et al., 2019). Hard plant tissues, such as bamboo and other stems, have little change in pore size before and after decellularization, or even become smaller; soft tissues, such as leaves, the structure becomes slack due to the long period of oscillation and washing during the decellularization process, resulting in the large increase of pore size. Lee et al. inoculated human induced pluripotent stem cells (HiPSCs) on several decellularized plant scaffolds, including apples, carrots, and sweet peppers. They found that only decellularized apple scaffolds could support the cell growth and reproduction, while the multiplication and growth of pluripotent stem cells on other scaffolds were poor, which indicated that the appropriate pore size is the key condition for supporting cell growth even if the scaffold components were similar (Lee et al., 2019).

**TABLE 3:** The pore size of representative plants before and after decellularization.

Plant type	Pore size (μm)	
	Before decellularization	After decellularization
Bamboo Fontana et al., 2017	81.1594	78.2609
Anthurium waro*c*queanum Fontana et al., 2017	40.5797	37.6812
Calathea zebrine Fontana et al., 2017	39.1305	84.0580
Orchid’s pseudobulb Fontana et al., 2017	63.7681	66.6667
Parsley Fontana et al., 2017	23.1884	237.826
Vanilla Fontana et al., 2017	86.9565	228.986
Apple Lee et al., 2019	321.951	/
Broccoli Lee et al., 2019	117.073	/
Jujube Lee et al., 2019	126.829	/
Carrot Lee et al., 2019	258.537	/
Persimmon Lee et al., 2019	473.171	/
Sweet pepper Lee et al., 2019	809.756	/

### Similarity in Vascular Network Between Plant and Human Tissues

At present, the lack of functional vascular tissue is an important obstacle to tissue engineering transplantation, which leads to poor prognosis of tissue after transplantation. Lee et al. found that although plants and animals used very different methods to deliver fluids and nutrients, their vascular networks were remarkably similar. Plant vascular systems follows Murray’s Law (McCulloh et al., 2003), which is also the physiological law describing the network design of the human cardiovascular system (Murray, 1926). In their study, the vascular network in the spinach leaves remained intact and could support the flow of red blood cells even after decellularization. By combining vascularized leaves with perfusion-based decellularization, natural vascularized scaffolds can be provided. However, Jansen et al. found that the vascular system of plant leaves might not be suitable for recellularization. Although cells can grow in the petiole, they cannot enter the vascular system of leaves through the petiole because the vascular is actually located in the parenchyma near the petiole and is not connected to the petiole cavity (Jansen et al., 2020). Therefore, although plant decellularized scaffolds have some similarities with human organs in general morphology, they still have slight differences, resulting in the fact that only simple-structured organs and tissues can be republicated. This is one of the disadvantages of plant decellularized scaffolds compared with animal decellularized scaffolds.

## Current Obstacles to the Application of Plant ECM

### Biodegradability of Plant Scaffolds *in vivo*

Cellulose is a kind of natural glucose linear polymer connected by 1,4-glucosidic bonds and hydrogen bonds. This is the reason why cellulose has high crystallinity, low solubility, and poor degradation *in vivo* (Miyamoto et al., 1989). Cellulose can be degraded by microorganisms and fungi in nature. However, because of the lack of appropriate enzymes to digest plant cellulose in mammals and humans, the degradation of decellularized plant scaffolds *in vivo* is also an urgent problem to be solved. Lee et al. (2019) found plant-derived scaffolding implants remained intact after 8 weeks.

At present, some studies have made preliminary progress in the degradation of cellulose *in vivo*. Entcheva et al. (2004) mixed cellulose scaffolds with cellulase (endoglucanase, exoglucanase, and β-glucosidase) for hydrolysis pretreatment, which can obtain different degrees of biodegradable and biocompatible cellulose scaffolds. Recently, Horn et al. (2012) found a new group of enzymes, classified as carbohydrate binding modules 33 (CBM33) and glycoside hydrolase 61 (GH61), which can catalyze the oxidative cleavage of polysaccharides. However, study has shown that cellulose enzymes have negative effect on the adhesion of some cells (Entcheva et al., 2004). The mechanical properties of plant scaffolds may be decreased during pre-hydrolysis and the degradation rate of the pretreated scaffolds *in vivo* is difficult to control due to the different degree of hydrolysis. In addition, whether the microstructural changes of the pre-hydrolyzed scaffolds will cause unexpected inflammatory responses *in vivo* is still unknown, which needs further study.

Another way to induce the degradability of cellulose is oxidation. Oxidized cellulose can be generated by various oxidizing agents, such as NaClO_2_ and CCl_4_ (Kumar and Yang, 2002; Zimnitsky et al., 2004; Saito et al., 2007). Oxidized cellulose can be degraded by hydrolysis, by mediated hydrolytic enzymes present in the serum supplement of cell culture media *in vitro* and *in vivo* (Dimitrijevich et al., 1990b). Aswathy et al. oxidized bamboo stems with different concentrations of sodium periodate. Sodium periodate can selectively cleavage the C2–C3 bond of the polysaccharide chain to convert hydroxyl group to dialdehyde. X-ray-diffraction (XRD) spectrum showed that the amorphous matrix of cellulose was increased and the crystallinity decreased after oxidation. Significant degradation of oxidized cellulose (70%) was observed after 7 days of subcutaneous implantation in mice compared with unoxidized scaffolds. At the same time, the oxidized cellulose is conducive to protein adsorption, thus promoting cell adhesion and its function. This may be due to the covalent bond between the amino group in the protein and the aldehyde group on the surface of the fiber (S H et al., 2021).

However, there are still several problems to be solved about the degradation of plant scaffolds. Although cellulose is the main component in plant ECM, degradation of other components (such as lignin and pectin) has not been studied yet, and the *in vivo* response of the entire decellularized plant tissue is also unclear.

### Immunogenicity of Plant Decellularized Scaffolds

Because of the relatively conservative nature of animal ECM, the main substances that cause immune rejection during the transplantation of animal-derived decellularized scaffolds are α-gal and MHC, which do not exist in plants. In contrast, different types and different parts (the rhizome and leaf) of plant extracellular matrix components, especially plant proteins, vary greatly, which makes it challenging to study the immunogenicity of plant scaffolds. Phan et al. performed THP-1 derived macrophage activation experiments to assess the potential of transgenic tobacco cell-derived extracellular matrix to induce TNF-α secretion as a readout for immune cell activation. Result shows that treating THP-1-derived macrophages with the BY-2 cell-derived matrices directly for 48 h resulted in increased TNF-α secretion, which may be induced by the phagocytosis of small particle. But the leachable components from the BY-2 cell-derived matrix, such as potential proteins, do not induce activations of macrophage (Phan et al., 2020). After subcutaneous embedding of oxygenated decellularized bamboos in mice, a large number of cell infiltration, especially macrophages, were observed, which might be related to the degradation of scaffolds. The oxidized scaffold may attract more macrophages due to its high hydrophilicity and amorphous properties. Macrophages secrete vascular endothelial growth factor, fibroblast growth factor, and platelet-derived growth factor, and initiate the process of vascular germination, anastomosis, and maturation, thus vascular formation can be seen around the scaffold, indicating its ability to facilitate the natural process of regeneration (S H et al., 2021). Modulevsky et al. implanted the cellulose scaffolds obtained from decellularized apples (proteins completely removed) under mice skin, and histological analysis revealed a typical foreign body reaction to the scaffold at 1 week. However, the immune response was observed to disappear gradually at 8 weeks. By 8 weeks, there was no immune response in the surrounding dermis tissue, and active fibroblast migration was observed in the cellulose scaffold (Modulevsky et al., 2016).

In general, the immune responses of acellular plant scaffolds *in vivo* have not been systematically evaluated. For example, immunohistochemical staining of macrophage phenotypes is necessary to understand its role in immune response and vascularization. M1 macrophages promote the killing of pathogens and increase the levels of cells associated with typical inflammatory symptoms, especially chronic inflammation. While M2 macrophages are considered as anti-inflammatory macrophages, which can promote immune regulation, tissue repair and tissue remodeling (Badylak and Gilbert, 2008).

#### Effects of Decellularization Protocol

Various protocols and reagents are used in the production of decellularized scaffolds to remove the nuclei and cellular content from tissues or organs while preserving the ECM structure. The decellularized scaffolds treated with detergents such as SDS were found to have cytotoxic effects due to the presence of residual SDS in the ECM (Caamaño et al., 2009). Therefore, thorough cleansing of the tissue is necessary to maintain the bioactivity of the acellular matrix (Syed et al., 2014). However, extensive cleaning procedures may reduce the mechanical properties of plant scaffolds and destroy the integrity of scaffolds. In addition, the Raman Spectroscopy and ATR-FTIR studies have shown that SDS has adverse effects on the ultrastructure of ECM in the process of decellularization of animal scaffolds, which may induce an immune response (Nara et al., 2016). The immune response may be triggered by exposure to the C-terminal, helical and N-terminal peptides of the collagen layer. But the effect of detergents on specific plant ECM is not yet clear due to the diversity of plant species.

#### Immunogenicity of Remaining Cell Debris

At present, a clear scientific basis for the decellularized plant scaffold criteria is still lacking. Studies at present have mainly adopted the standard of animal decellularized scaffolds: DNA content of <50 ng double-stranded DNA/mg ECM (Chakraborty et al., 2020). Generally, Safranin and Fast Green are required to confirm the complete elimination of nucleus.

#### Immunogenicity of Plant Protein

During their long evolutionary histories, the sequences and structures of the proteins of plants and animals have become quite different. Important components of animal ECMs are proteoglycans (PGs), which is formed by covalently linking one or more glycosaminoglycans (GAGs) chains on a core protein (Frantz et al., 2010; Kular et al., 2014; American Association of Neurological Surgeons et al., 2018). Animal extracellular matrixes also have a large content of proteins, such as collagens, elastin, fibronectin, laminins, and glycoproteins. In the plant extracellular matrix, however, proteins are merely minute components of the plant cell wall and are trapped in the complex network of polysaccharides. Due to the diversity of plant species and the limitations of plant protein extraction, it is estimated that only a few proteins (less than 5%) have clear biochemical activity, localization, and biological effects (Albenne et al., 2014; Niehaus et al., 2015).

Some plant proteins, mainly cereal proteins, have been used to construct scaffolds (Reddy and Yang, 2011). Soy protein films crosslinked with glyoxal and tannic acid were studied as potential membrane materials and have been proved to have good biocompatibility *in vitro* (Silva et al., 2003). However, some have been proved to be immunogenetic, such as zein and sericin (Altman et al., 2003; Hurtado-López and Murdan, 2006). Hence, the potential immunogenicity of plant protein-based materials needs further study. First, analysing the protein compositions and content of plants before and after decellularization is a must, which is not available in most current studies. Second, conducting a comprehensive evaluation of the bioactive proteins abundant in specific plant tissue, including the evaluation of biocompatibility and bioactivity. T-cell proliferation and pluripotent myeloid stem cell (PMSC) assay can be used to determine the cytocompatibility of plant proteins. PMSC assay co-cultures the protein with human peripheral blood mononuclear cells (PBMCs) and investigate the upregulation of dendritic cells (DC) maturation markers, such as CD83, CD80, and CD86. For different bioactive plant proteins, targeted *in vitro* experiments can be carried out. Then, moderately retaining the identified substances in plant tissues to obtain a bioactive plant scaffold, by adjusting the decellularization protocol (e.g., by reducing the concentration of detergent or shortening the decellularization time), may be a possible way to obtain a bioactive plant scaffold.

## Application of Plant Decellularized ECM

### Recellularization of Decellularized Scaffold

**FIGURE 3:**
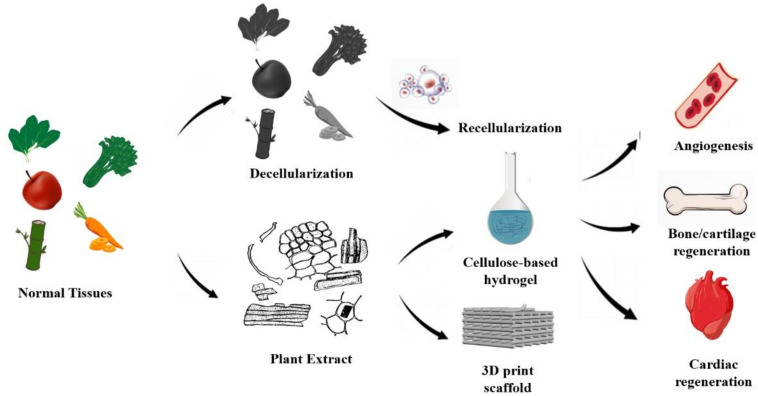
Preparation and application of decellularized plant scaffolds: Current bioactive plant-derived scaffolds (1) recellularization of decellularized plant scaffolds, (2) plant extracts were used for cardiovascular regeneration and bone tissue regeneration by gelation and 3D printing.

The good adhesion, proliferation, and function of human cells on the plant ECM scaffold determine the successful application of decellularized plant tissues in human tissue culture. At present, decellularized plant scaffolds mainly recellularized with embryonic stem cells or mesenchymal stem cells, which have a good prospect in cardiovascular regeneration biology and bone tissue regeneration biology (Gershlak et al., 2017; Fontana et al., 2017; Table 4 and Figure 3). Fontana et al. (2017) planted human mesenchymal stem cells (MSCs) and human skin fibroblasts (hDFs), respectively, after biomineralization and tripeptide Arginine-Glycine-Aspartate (RGD) modification of parsley stem cells. It was observed that MSCs and hDFs could proliferate greatly at the same time (Fontana et al., 2017), and Gershlak et al. (2017) seeded human umbilical vein endothelial cells and human pluripotent stem-cell derived cardiomyocytes on the decellularized spinach scaffold modified with fibronectin. It was observed that the adherent human umbilical vein endothelial cells (HUVECs) arranged with the inner vessel wall and survived; within 21 days, the spontaneous contraction of embryonic stem cell derived cardiomyocytes (HPS-CMs) was observed when it attached to the surface of the leaf scaffold.

**TABLE 4:** Review of plant scaffold recellularization protocols.

Plant type	Cell type	Research Purpose	Result	References
Apple	Human epithelial cells	Adipogenic differentiation	The intracellular lipid accumulation typical of adipocytes is observed	Silva et al. (2003)
	3T3-L1 murine cell line (ECACC No 86052701)			
	Induced pluripotent stem cells	Osteoblastic differentiation	Mineralizing nodules are observed, osteogenic markers (OCN, COL-1) are detected	Harris et al. (2021)
*Spinach* leaf	Prostate cancer cells	Tumor cell response to the decellularized spinach leaf scaffolds	YAP/TAZ signaling downregulation, cellular morphology alteration, and proliferation rate decrease	Lacombe et al. (2020)
	Melanoma cells			
	Human umbilical vein endothelial cells	Leaf vasculature endothelialisation	Remain viable and adhered	Gershlak et al. (2017)
	Human pluripotent stem cell- derived cardiomyocytes	Function examination	Spontaneously contract at Day 5	Gershlak et al. (2017)
Carrot	Pre-osteoblast cell (MC3T3-E1)	Osteogenic differentiation	Nano-sized particles are detected on the surface of differentiation-induced cells	Contessi Negrini et al. (2020)
Green onion	Human skeletal myoblasts (HSMCs)	Muscle differentiation	Support growth, proliferation, and differentiation of human skeletal muscle and provide the necessary alignment of myotubes required for enhanced functional contractility of muscle tissue	Cheng et al. (2020)
Bamboo	Mesenchymal stem cells	Osteogenic differentiation	High alkaline phosphatase activity and osteocalcin release	S H et al. (2021)

However, because of the removal of the most part of extracellular matrix and the lack of cell adhesion molecules, the obtained cellulose scaffolds perform poorly in cell adhesion and have to be modified with some amino acids to support cell growth, such as RGD and poly-L-lysine (PLL) (Fontana et al., 2017). This limitation can also be attributed to the lack of research on plant extracellular matrix and plant proteins, as mentioned above. In addition, since the plant scaffolds lack the environment or the corresponding cytokines to induce human cell differentiation, covering the plant decellularized scaffolds with a differentiation inducing coating may be the current research direction, such as vascular-induced differentiation or bone-induced differentiation.

### Decellularized Plant Scaffold for Cartilage and Bone Tissue Construction

Decellularized plant scaffolds have not been reported for cartilage engineering, but some plant extracts have been proved to have the potential to promote cartilage formation. In 2018, a plant-derived polysaccharide carboxymethyl cellulose (CMC) was sulfated and combined with previous research results to prepare a macroporous framework for injection (Waghmare et al., 2018). With its unique shape memory characteristics and its adhesion to growth factors, the scaffold effectively simulates the complex process of natural cartilage regeneration in cartilage repair.

Decellularized plant scaffolds have a good prospect in promoting osteogenesis. Lee et al. planted pluripotent stem cells into decellularized apple scaffolds. After 96 h of culture, the surviving cells still expressed stem cell markers (OCT3/4, Sox2, Nanog, Lin28, dppb5, tdgf1, and ssea4), and their levels were similar to those of iPSCs cultured in 2D medium, which meant that HiPSCs retained pluripotency in the scaffolds. After 3 weeks of culture in the osteogenic induction medium, the pluripotent stem cells on the decellularized apple scaffold showed obvious osteogenic differentiation. In addition, after the transplantation into the rat skull defect model, the scaffold significantly promoted the healing of defects *in vivo*, and had the potential to support vascular growth for bone regeneration (Lee et al., 2019). However, in the current research, decellularized plant scaffolds only provide a porous three-dimensional growth space for cells, and the scaffold itself does not directly promote bone healing function. At present, some plant extracts have been found to have the potential to promote osteogenesis and to regulate immune response. For example, garlic contains a large number of pharmaceutically active sulfur compounds, which can reduce the clinical symptoms of gingivitis (Rodrigues and Percival, 2019); a paste prepared from quinoa leaves, which have a large content of saponins, flavonoids, tannins, and alkaloids, may contribute to its effect on bone regeneration (Pinheiro Neto et al., 2017). Therefore, constructing natural decellularized scaffolds with osteogenic activity will be the future research direction.

### Decellularized Plant Scaffold for Muscle Regeneration

Some common fruits or vegetables with unique architectures are suited for muscle alignment. Since voluntary striated muscle fibers are arranged in bundles parallel to each other in human body, plant scaffolds with anisotropic structures when cut longitudinally versus transverse are required, such as leek, asparagus, green onion and celery (Cheng et al., 2020). Cheng et al. (2020) seeded human skeletal muscle cells on green onion-derived cellulose scaffolds. Cells aligned perfectly on the outer white bulb-derived cellulose scaffolds after 6 days. They also found that repeating grooves with 20 μm wide, 10 μm deep, and 5 μm spacing seems to be the most appropriate topography to generate an aligned muscle cell monolayer (Cheng et al., 2020). However, the study of decellularized plant scaffolds for muscle regeneration are limited to *in vitro* at present, and a large number of studies are needed for future application *in vivo*, such as how to achieve the maximum differentiation of muscle cells on the scaffold, and how to ensure that the synthesized skeletal muscle tissue has sufficient contractile strength.

## Conclusion

Plant scaffold not only has the characteristics of animal architecture but also provides the 3D environment and vascular system needed by stem cells. Moreover, it has good modifiability, which makes it suitable for the proliferation and differentiation of a variety of stem cells and can promote the development and differentiation of blood vessels. Nano scale fine structure plant framework is used for *in vivo* organ or tissue regeneration and *in vitro* tissue regeneration research and clinical application, which provides rare precise organ tissue engineering materials.

The application of decellularized plant scaffold is still in its infancy. The characteristics of plant tissue materials lay a foundation for its application. (1) The diversity of plant structure and composition allows for the construction of decellularized scaffolds suitable for different organs. (2) A plant framework has many advantages over an animal framework; it is characterized by good modification, no pollution or pathogens, a low cost and a simple production process, making it suitable for large-scale production and basic research. (3) The striking similarity between plant tissue and some organs of the human body promise its good prospect for application *in vivo* and *in vitro*. However, many challenges still need to be overcome in the clinical application of decellularized plant scaffolds. (1) The existing decellularization methods will greatly damage the structure of plants, including the increase of pores and the decrease of mechanical properties. Therefore, the decellularization methods suitable for plants, especially for some fragile tissues, are in urgent need of research. (2) Due to the lack of cellulase in animals, plant scaffolds can hardly be degraded *in vivo* and the existing methods may have negative effects on the scaffolds. (3) The evaluation methods of the biocompatibility of plant scaffolds are not clear yet, and there are few studies on the immune process caused by plant scaffolds *in vivo*. (4) The current decellularized plant scaffolds only provide a three-dimensional scaffold for cell growth, but their biological activity is poor. Therefore, it is a feasible research direction to endow the scaffold with corresponding biological activity by coating and other methods.

## Author Contributions

All authors listed have made a substantial, direct and intellectual contribution to the work, and approved it for publication.

## Conflict of Interest

The authors declare that the research was conducted in the absence of any commercial or financial relationships that could be construed as a potential conflict of interest.

## References

[B1] Adamski M. Fontana G. Gershlak J. R. Gaudette G. R. Le H. D. Murphy W. L. (2018). Two methods for decellularization of plant tissues for tissue engineering applications. *J. Vis. Exp.* 1:57586. 10.3791/57586 29912197 PMC6101437

[B2] Albenne C. Canut H. Hoffmann L. Jamet E. (2014). Plant cell wall proteins: a large body of data, but what about runaways? *Proteomes* 2 224–242. 10.3390/proteomes2020224 28250379 PMC5302738

[B3] Altman G. H. Diaz F. Jakuba C. Calabro T. Horan R. L. Chen J. (2003). Silk-based biomaterials. *Biomaterials* 24 401–416. 10.1016/s0142-9612(02)00353-8 12423595

[B4] Author Anonymous (2021). OPTN/SRTR. *Am. J. Transplant.* 21 (Suppl 2) 11–20.^∗^ 10.1111/ajt.16493 33595194

[B5] Badylak S. F. Gilbert T. W. (2008). Immune response to biologic scaffold materials. *Semin. Immunol.* 20 109–116. 10.1016/j.smim.2007.11.003 18083531 PMC2605275

[B6] Badylak S. F. Freytes D. O. Gilbert T. W. (2009). Extracellular matrix as a biological scaffold material: Structure and function. *Acta Biomater.* 5 1–13. 10.1016/j.actbio.2008.09.013 18938117

[B7] Bush J. R. Liang H. Dickinson M. Botchwey E. A. (2016). Xylan hemicellulose improves chitosan hydrogel for bone tissue regeneration. *Polym Adv. Technol.* 27 1050–1055. 10.1002/pat.3767 27587941 PMC5004929

[B8] Caamaño S. Shiori A. Strauss S. H. Orton E. C. (2009). Does sodium dodecyl sulfate wash out of detergent-treated bovine pericardium at cytotoxic concentrations? *J. Heart Valve Dis.* 18 101–105. 19301560

[B9] Caffall K. H. Mohnen D. (2009). The structure, function, and biosynthesis of plant cell wall pectic polysaccharides. *Carbohydr. Res.* 344 1879–1900. 10.1016/j.carres.2009.05.021 19616198

[B10] Chakraborty J. Roy S. Ghosh S. (2020). Regulation of decellularised matrix mediated immune response. *Biomater. Sci.* 8 1194–1215. 10.1039/c9bm01780a 31930231

[B11] Cheng Y. W. Shiwarski D. J. Ball R. L. Whitehead K. A. Feinberg A. W. (2020). Engineering aligned skeletal muscle tissue using decellularized plant-derived scaffolds. *ACS Biomater. Sci. Eng.* 6 3046–3054. 10.1021/acsbiomaterials.0c00058 33463300 PMC8628848

[B12] Contessi Negrini N. Toffoletto N. Farè S. Altomare L. (2020). Plant tissues as 3d natural scaffolds for adipose, bone and tendon tissue regeneration. *Front. Bioeng. Biotechnol.* 8:723. 10.3389/fbioe.2020.00723 32714912 PMC7344190

[B13] Czaja W. K. Young D. J. Kawecki M. Brown R. M. Jr. (2007). The future prospects of microbial cellulose in biomedical applications. *Biomacromolecules* 8 1–12. 10.1021/bm060620d 17206781

[B14] Czaja W. Krystynowicz A. Bielecki S. Brown R. M. (2006). Microbial cellulose–the natural power to heal wounds. *Biomaterials* 27 145–151. 10.1016/j.biomaterials.2005.07.035 16099034

[B15] Dimitrijevich S. D. Tatarko M. Gracy R. W. Wise G. E. Oakford L. X. Linsky C. B. (1990b). *in vivo* degradation of oxidized, regenerated cellulose. *Carbohydr. Res.* 198 331–341. 10.1016/0008-6215(90)84303-c 2379193

[B16] Entcheva E. Bien H. Yin L. Chung C. Y. Farrell M. Kostov Y. (2004). Functional cardiac cell constructs on cellulose-based scaffolding. *Biomaterials.* 25 5753–5762. 10.1016/j.biomaterials.2004.01.024 15147821

[B17] Fang H. Y. Chen Y. K. Chen H. H. Lin S. Y. Fang Y. T. (2012). Immunomodulatory effects of feruloylated oligosaccharides from rice bran. *Food Chem.* 134 836–840. 10.1016/j.foodchem.2012.02.190 23107698

[B18] Fernandes A. N. Thomas L. H. Altaner C. M. Callow P. Forsyth V. T. Apperley D. C. (2011). Nanostructure of cellulose microfibrils in spruce wood. *Proc. Natl Acad. Sci. USA* 108 E1195–E1203. 10.1073/pnas.1108942108 22065760 PMC3223458

[B19] Fontana G. Gershlak J. Adamski M. Lee J. S. Matsumoto S. Le H. D. (2017). Biofunctionalized Plants as Diverse Biomaterials for Human Cell Culture. *Adv. Healthc. Mater.* 6:10.1002/adhm.201601225. 10.1002/adhm.201601225 PMC5490445 28319334

[B20] Frantz C. Stewart K. M. Weaver V. M. (2010). The extracellular matrix at a glance. *J. Cell Sci.* 123(Pt 24) 4195–4200. 10.1242/jcs.023820 21123617 PMC2995612

[B21] From the American Association of Neurological Surgeons (Aans), American Society of Neuroradiology (Asnr), Cardiovascular and Interventional Radiology Society of Europe (Cirse), Canadian Interventional Radiology Association (Cira), Congress of Neurological Surgeons (Cns), European Society of Minimally Invasive Neurological Therapy (Esmint) (2018). Multisociety consensus quality improvement revised consensus statement for endovascular therapy of acute ischemic stroke. *Int. J. Stroke* 13 612–632. 10.1177/1747493018778713 29786478

[B22] Gershlak J. R. Hernandez S. Fontana G. Perreault L. R. Hansen K. J. Larson S. A. (2017). Crossing kingdoms: Using decellularised plants as perfusable tissue engineering scaffolds. *Biomaterials* 125 13–22. 10.1016/j.biomaterials.2017.02.011 28222326 PMC5388455

[B23] Guler S. Aslan B. Hosseinian P. Aydin H. M. (2017). Supercritical carbon dioxide-assisted decellularization of aorta and cornea. *Tissue Eng. Part C Methods.* 23 540–547. 10.1089/ten.TEC.2017.0090 28726559

[B24] Harris A. F. Lacombe J. Liyanage S. Han M. Y. Wallace E. Karsunky S. (2021). Supercritical carbon dioxide decellularization of plant material to generate 3D biocompatible scaffolds. *Sci. Rep.* 11 3643. 10.1038/s41598-021-83250-9 33574461 PMC7878742

[B25] Hori R. Muller M. Watanabe U. Lichtenegger H. C. Fratzl P. Sugiyama J. (2002). The importance of seasonal differences in the cellulose microfibril angle in softwoods in determining acoustic properties. *J. Mater. Sci.* 37 4279–4284. 10.1023/A:1020688132345

[B26] Horn S. J. Vaaje-Kolstad G. Westereng B. Eijsink V. G. (2012). Novel enzymes for the degradation of cellulose. *Biotechnol. Biofuels.* 5:45. 10.1186/1754-6834-5-45 22747961 PMC3492096

[B27] Huerta S. Varshney A. Patel P. M. Mayo H. G. Livingston E. H. (2016). Biological mesh implants for abdominal hernia repair: us food and drug administration approval process and systematic review of its efficacy. *JAMA Surg.* 151 374–381. 10.1001/jamasurg.2015.5234 26819222

[B28] Hurtado-López P. Murdan S. (2006). An investigation into the adjuvanticity and immunogenicity of zein microspheres being researched as drug and vaccine carriers. *J. Pharm. Pharmacol.* 58 769–774. 10.1211/jpp.58.6.0007 16734978

[B29] Jansen K. Evangelopoulou M. Pou Casellas C. Abrishamcar S. Jansen J. Vermonden T. (2020). Spinach and chive for kidney tubule engineering: the limitations of decellularized plant scaffolds and vasculature. *AAPS J.* 23:11. 10.1208/s12248-020-00550-0 33369701 PMC7769781

[B30] Johnson M. (2013). Detergents: tTriton X-100, tTween-20, and mMore. *Mater. Methods* 3:163. 10.13070/mm.en.3.163

[B31] Kular J. K. Basu S. Sharma R. I. (2014). The extracellular matrix: sStructure, composition, age-related differences, tools for analysis and applications for tissue engineering. *J. Tissue Eng.* 5:2041731414557112. 10.1177/2041731414557112 25610589 PMC4883592

[B32] Kumar V. Yang D. (2002). HNO3/H3PO4–NANO2 mediated oxidation of cellulose-preparation and characterization of bioabsorbable oxidized celluloses in high yields and with different levels of oxidation. *Carbohydr. Polym.* 48 403–412. 10.1016/s0144-8617(01)00290-9

[B33] Lacombe J. Harris A. F. Zenhausern R. Karsunsky S. Zenhausern F. (2020). Plant-based scaffolds modify cellular response to drug and radiation exposure compared to standard cell culture models. *Front. Bioeng. Biotechnol.* 8:932. 10.3389/fbioe.2020.00932 32850759 PMC7426640

[B34] Lee J. Jung H. Park N. Park S. H. Ju J. H. (2019). Induced osteogenesis in plants decellularised scaffolds. *Sci. Rep.* 9:20194. 10.1038/s41598-019-56651-0 PMC6934596 31882858

[B35] Liuyun J. Yubao L. Chengdong X. (2009). Preparation and biological properties of a novel composite scaffold of nanohydroxyapatite/chitosan/carboxymethyl cellulose for bone tissue engineering. *J. Biomed. Sci.* 16:65. 10.1186/1423-0127-16-65 19594953 PMC2720940

[B36] Mathew A. P. Oksman K. Pierron D. Harmand M. F. (2012). Fibrous cellulose nanocomposite scaffolds prepared by partial dissolution for potential use as ligament or tendon substitutes. *Carbohydr. Polym.* 87 2291–2298. 10.1016/j.carbpol.2011.10.063

[B37] McCulloh K. A. Sperry J. S. Adler F. R. (2003). Water transport in plants obeys Murray’s law. *Nature.* 421 939–942. 10.1038/nature01444 12607000

[B38] Miyamoto T. Takahashi S. Ito H. Inagaki H. Noishiki Y. (1989). Tissue biocompatibility of cellulose and its derivatives. *J. Biomed. Mater. Res.* 23 125–133. 10.1002/jbm.820230110 2708402

[B39] Modulevsky D. J. Cuerrier C. M. Pelling A. E. (2016). Biocompatibility of Subcutaneously Implanted Plant-Derived Cellulose Biomaterials. *PLoS One* 11:e0157894. 10.1371/journal.pone.0157894 27328066 PMC4915699

[B40] Modulevsky D. J. Lefebvre C. Haase K. Al-Rekabi Z. Pelling A. E. (2014). Apple derived cellulose scaffolds for 3D mammalian cell culture. *PLoS One* 9:e97835. 10.1371/journal.pone.0097835 24842603 PMC4026483

[B41] Müller F. A. Müller L. Hofmann I. Greil P. Wenzel M. M. Staudenmaier R. (2006). Cellulose-based scaffold materials for cartilage tissue engineering. *Biomaterials* 27 3955–3963. 10.1016/j.biomaterials.2006.02.031 16530823

[B42] Murray C. D. (1926). The physiological principle of minimum work: i. the vascular system and the cost of blood volume. *Proc. Natl. Acad. Sci. U.S.A.* 12 207–214. 10.1073/pnas.12.3.207 16576980 PMC1084489

[B43] Nara S. Chameettachal S. Midha S. Murab S. Ghosh S. (2016). Preservation of biomacromolecular composition and ultrastructure of a decellularized cornea using a perfusion bioreactor. *RSC Adv.* 6 2225–2240. 10.1039/C5RA20745B

[B44] Niehaus T. D. Thamm A. M. de Crécy-Lagard V. Hanson A. D. (2015). Proteins of unknown biochemical function: a persistent problem and a roadmap to help overcome it. *Plant Physiol.* 169 1436–1442. 10.1104/pp.15.00959 26269542 PMC4634069

[B45] Peng X. Y. Hu M. Liao F. Yang F. Ke Q. F. Guo Y. P. (2019). La-Doped mesoporous calcium silicate/chitosan scaffolds for bone tissue engineering. *Biomater. Sci.* 7 1565–1573. 10.1039/c8bm01498a 30688345

[B46] Peura M. Muller M. Vainio U. Saren M.-P. Saranpaa P. Serimaa R. (2008). X-ray microdiffraction reveals the orientation of cellulose microfibrils and the size of cellulose crystallites in single Norway spruce tracheids. *Trees* 22 49–61. 10.1007/s00468-007-0168-5

[B47] Phan N. V. Wright T. Rahman M. M. Xu J. Coburn J. M. (2020). *in vitro* biocompatibility of decellularized cultured plant cell-derived matrices. *ACS Biomater. Sci. Eng..* 6 822–832. 10.1021/acsbiomaterials.9b00870 33464854

[B48] Pinheiro Neto V. F. Ribeiro R. M. Morais C. S. Campos M. B. Vieira D. A. Guerra P. C. (2017). Chenopodium ambrosioides as a bone graft substitute in rabbits radius fracture. *BMC Complement. Altern. Med.* 17:350. 10.1186/s12906-017-1862-5 28676049 PMC5496593

[B49] Ponnusamy V. K. Nguyen D. D. Dharmaraja J. Shobana S. Banu J. R. Saratale R. G. (2019). A review on lignin structure, pretreatments, fermentation reactions and biorefinery potential. *Bioresour. Technol.* 271 462–472. 10.1016/j.biortech.2018.09.070 30270050

[B50] Reddy N. Yang Y. (2011). Potential of plant proteins for medical applications. *Trends Biotechnol.* 29 490–498. 10.1016/j.tibtech.2011.05.003 21665302

[B51] Rodrigues C. Percival S. S. (2019). Immunomodulatory effects of glutathione, garlic derivatives, and hydrogen sulfide. *Nutrients* 11:295. 10.3390/nu11020295 30704060 PMC6412746

[B52] S H A. Mohan C. C. P S U. Krishnan A. G. Nair M. B. (2021). Decellularization and oxidation process of bamboo stem enhance biodegradation and osteogenic differentiation. *Mater. Sci. Eng. C Mater. Biol. Appl.* 119:111500. 10.1016/j.msec.2020.111500 33321600

[B53] Saito T. Kimura S. Nishiyama Y. Isogai A. (2007). Cellulose nanofibers prepared by TEMPO-mediated oxidation of native cellulose. *Biomacromolecules* 8 2485–2491. 10.1021/bm0703970 17630692

[B54] Seba A. (1730). III The anatomical preparation of vegetables, by Albertus Seba, F. R. S. Communicated to the Royal Society by Sir Hans Sloane, Bart. Pr. R. S. and Col. Med. Lond. Translated from the German, by Mr. Zolman, F. R. SPhil. *Trans. R. Soc.* 36 441–444. 10.1098/rstl.1729.0061

[B55] Silva G. A. Vaz C. M. Coutinho O. P. Cunha A. M. Reis R. L. (2003). *in vitro* degradation and cytocompatibility evaluation of novel soy and sodium caseinate-based membrane biomaterials. *J. Mater. Sci. Mater. Med.* 14 1055–1066. 10.1023/b:jmsm.0000004002.11278.30 15348498

[B56] Syed O. Walters N. J. Day R. M. Kim H. W. Knowles J. C. (2014). Evaluation of decellularization protocols for production of tubular small intestine submucosa scaffolds for use in oesophageal tissue engineering. *Acta Biomater.* 10 5043–5054. 10.1016/j.actbio.2014.08.024 25173840

[B57] Waghmare N. A. Arora A. Bhattacharjee A. Katti D. S. (2018). Sulfated polysaccharide mediated TGF-β1 presentation in preformed injectable scaffolds for cartilage tissue engineering. *Carbohydr Polym.* 193 62–72. 10.1016/j.carbpol.2018.03.091 29773398

[B58] Wegst U. G. Bai H. Saiz E. Tomsia A. P. Ritchie R. O. (2015). Bioinspired structural materials. *Nat. Mater.* 14 23–36. 10.1038/nmat4089 25344782

[B59] Zablackis E. Huang J. Müller B. Darvill A. G. Albersheim P. (1995). Characterization of the cell-wall polysaccharides of Arabidopsis thaliana leaves. *Plant Physiol.* 107 1129–1138. 10.1104/pp.107.4.1129 7770522 PMC157245

[B60] Zaborowska M. Bodin A. Bäckdahl H. Popp J. Goldstein A. Gatenholm P. (2010). Microporous bacterial cellulose as a potential scaffold for bone regeneration. *Acta Biomater.* 6 2540–2547. 10.1016/j.actbio.2010.01.004 20060935

[B61] Zhang Y. Jiang M. Zhang Y. Cao Q. Wang X. Han Y. (2019). Novel lignin-chitosan-PVA composite hydrogel for wound dressing. *Mater. Sci. Eng. C Mater. Biol. Appl.* 104:110002. 10.1016/j.msec.2019.110002 31499949

[B62] Zhou S. Liu X. Guo Y. Wang Q. Peng D. Cao L. (2010). Comparison of the immunological activities of arabinoxylans from wheat bran with alkali and xylanase-aided extraction. *Carbohydrate Polymers.* 81 784–789. 10.1016/j.carbpol.2010.03.040

[B63] Zimnitsky D. S. Yurkshtovich T. L. Bychkovsky P. M. (2004). Synthesis and characterization of oxidized cellulose. *J. Polym. Sci. A Polym. Chem.* 42 4785–4791.

